# Cellular exposure to muscle relaxants and propofol could lead to genomic instability *in vitro*

**DOI:** 10.1016/S1674-8301(12)60021-9

**Published:** 2012-03

**Authors:** Allen Edward Coleman, Nicole McNeil, Alexander Leonidovich Kovalchuck, Dara Wangsa, Thomas Ried, Hong Wang

**Affiliations:** a Department of Anesthesiology, Wayne State University, Detroit Medical Center, Detroit, Michigan 48201, USA;; b Laboratory of Cancer Genomics, National Cancer Institute, National Institutes of Health, Bethesda, Maryland 20892, USA;; c Laboratory of Immunogenetics, National Institute of Allergy and Infectious Disease, National Institutes of Health, Rockville, Maryland 20852, USA.

**Keywords:** spectral karyotyping (SKY), genomic instability, epigenetic, P388, propofol, cisatracurium, vecuronium, pancuronium, aneuploidy

## Abstract

Anesthesia is widely used in several medical settings and accepted as safe. However, there is some evidence that anesthetic agents can induce genomic changes leading to neural degeneration or apoptosis. Although chromosomal changes have not been observed *in vivo*, this is most likely due to DNA repair mechanisms, apoptosis, or cellular senescence. Potential chromosomal alterations after exposure to common anesthetic agents may be relevant in patients with genomic instability syndromes or with aggressive treatment of malignancies. In this study, the P388 murine B cells were cultured *in vitro*, and spectral karyotyping (SKY) was utilized to uncover genome-wide changes. Clinically relevant doses of cisatracurium and propofol increased structural and numerical chromosomal instability. These results may be relevant in patients with underlying chromosomal instability syndromes or concurrently being exposed to chemotherapeutic agents. Future studies may include utilization of stimulated peripheral blood lymphocytes to further confirm the significance of these results.

## INTRODUCTION

Anesthesia is accepted as having an excellent safety record[1]. However, several studies have implicated common anesthetic agents in inducing genomic changes *in vitro* or damaging cells and initiating programmed cell death[2]-[5]. Recent studies have demonstrated an increase in the rate of neural degeneration[6],[7] in developing animals which were exposed *in utero* to volatile anesthetics, gamma-aminobutyric acid (GABA) agonists, or *N*-methyl-*D*-aspartate (NMDA) antagonists. Furthermore, young children exposed to multiple anesthetic agents have been found to be at increased risk for developing learning disabilities[8].

Although the occurrence of chromosomal changes following anesthesia has not been observed *in vivo*[9], such aberrations are likely transient and eliminated by apoptosis, genomic repair, or cellular senescence. Patients with genomic instability syndromes or receiving chemotherapy or radiation therapy may in the presence of inciting agents have increased risk of either new sporadic cancer or altering clonality of existing malignancy. Our study aimed to assess the genomic effects of common anesthetic drugs by utilizing the P388 lymphoma cell line, a previously characterized stable murine B cell line[10], and spectral karyotyping (SKY), a fluorescent *in situ* hybridization methodology that allows unique full color chromosomal painting[11],[12].

## MATERIALS AND METHODS

### Anesthetic drugs

Cisatracurium at doses of 0.2 and 2.0 µg/kg; pancuronium at doses of 0.1 and 1.0 µg/kg; propofol at doses of 2.0 and 20 µg/kg; vecuronium at doses of 0.1 and 1.0 µg/kg. Untreated P388 lymphoma cells were used as the control.

### Cell culture and metaphase preparation

P388 lymphoma cells were cultured for 6 h in the presence of the drug at two different doses or untreated control in RPMI media supplemented with 10% fetal calf serum (FCS), 200 mmol/L *L*-glutamine, and 50 µmol/L 2-mercaptoethanol at 37°C in a humidified incubator containing 5% CO_2_ in air. Cells were incubated in hypotonic 75 mmol/L KCl at 37°C for 15 min, followed by fixation in methanol/acetic acid (3:1 *V/V*). Chromosomes were prepared by dropping fixed cells onto humidified slides to facilitate chromosomal spreading.

The metaphase slides to be used for FISH were denatured in preparation for hybridization by adding 70% formamide/2×SSC (70 µL deionized formamide, 3 µL 20×SSC, 27 µL sterile water, pH 7.5) to a 24 mm×60 mm coverslip. The slides were gently touched to the coverslip, and then placed on a slide warmer that was pre-heated at 80°C. The slides were removed after 1 min and immediately dehydrated in an ethanol series. The slides were air dried in preparation of probe application. The SKY probe was denatured for 5 min at 80°C followed by a pre-annealing step for 1-2 h at 37°C. The probe was then applied to the slides and hybridized for 24-72 h at 37°C.

### SKY preparation and analysis

SKY in mouse, described in detail elsewhere[11],[12], utilizes 20 uniquely labeled chromosome fluorescent *in situ* hybridization (FISH) probes, fluorescent microscopy, digital imaging, and Fourier spectroscopy to visualize each chromosome in a different color. DNA probes are obtained by flow sorting of individual mouse chromosomes, followed by amplification of chromosomal DNA by two rounds of degenerate oligonucleotide-primed PCR, and labeling of the PCR product in a combinatorial manner with direct fluorochromes (Spectrum Green and Orange, Vysis; Texas Red, Molecular Probes) and indirectly with digoxin and biotin (Roche Applied Science, Indianapolis, IN, USA).

The metaphase slides to be used for FISH and the SKY probe were prepared as described above. The probe was then applied to the slides and hybridized for 24-72 h at 37°C. Slides were incubated for 72 h at 37°C, washed in salt solution at 45°C, and counter stained with 4′,6-diamidino-2-phenylindole (DAPI, Sigma, St. Louis, MO, USA). The images were acquired on a microscope fitted with an interferometer and CCD camera. Image analysis was performed by Fourier transformation, a mathematical retrieval of combinatorial labeled fluorescent signals.

In order to visualize the hybridization of the SKY probe on the slides, post-hybridization washes were performed with three times of washes in 50% formamide, 2×SSC at 45°C and three times of washes in 1×SSC at 45°C. The biotinylated probe sequences were detected by incubation with avidin Cy5, and the digoxin-labeled probe sequences were detected with a mouse anti-digoxin antibody and sheep Cy5.5 antimouse antibody (Amersham Pharmacia Biotech, Piscataway, NJ, USA). The slides were then washed in 4×SSC/Tween and dehydrated in an ethanol series, followed by counterstaining with DAPI. Finally, antifade (or 1,4-phenylene-diamine, Sigma) was applied to each slide to prevent photobleaching. The slides were stored in a cardboard folder at 4°C until imaging. The hybridized slides were illuminated by a Xenon lamp (OptiQuip770/1600), and the light emitted from each point of the sample was collected by the microscope objective and directed to a custom designed triple-band pass optical filter (Chroma Technology, Brattleboro, VT, USA). The filter was designed for the simultaneous excitement of all dyes and the measurement of their emission spectra in a single exposure time. The light collected by this filter was transferred to a Sagnac interferometer within a SD200 SpectraCube (Applied Spectral Imaging, Carlsbad, CA, USA) on an inverted DMIRBE microscope (Leica, Wetzlar, Germany). A Fourier transformation spectrometer within the optical head of the microscope measured the emission spectrum of light, and a CCD camera captured the images that are processed by a personal computer. The emission spectra can be converted to display colors (also called, pseudocolor images) by adding red, green, and blue to different ranges in the spectrum. This was done using the Spectral Imaging program (Applied Spectral Imaging, Carlsbad, CA, USA). Spectral classification colors were generated by a pixel-to-pixel measurement of the spectrum. Pixels with the same spectra were assigned the same classification color and produced a unique chromosome color to distinguish all chromosomes from each other. Further analysis and karyotyping were conducted with the Applied Spectral Imaging Software (Vista, CA, USA).

## RESULTS

### Aminosteroid muscle relaxants

Both the vecuronium and pancuronium-treated cells at clinically relevant dosages displayed stability similar to control, and the modal number of chromosomes was 39 and there were no new chromosomal aberrations (***Table 1***). However, some cells treated with vecuronium had tetraploid cells (3/20) containing 79, 79, and 80 chromosomes, respectively.

**Table 1:** Inducible chromosomal aberrations detected by spectral karyotyping

Image^a^	CisA	CisB	PanA	PanB	VecA	VecB	ProA	ProB
1	40	40 T(5;19)	39	41 Dmin	40	40	37 Dmin	39
2	40 T(2;10)	36	39	78*	80	38	40 Rb(5.16)	80
3	40	36 Dmin	39	80* 2CB, ECE	43	39	39 ECE	39 Dmin Rb(8.10)
4	39	38	37	40	37	98*	37	84 DM
5	39	38	39	38	39	76	38	39
6	38	41	37	41	39	80	39	39
7	41 T(10;19) Is(l;5)	78*	38	40	37	39	38 Rb(14.16)T(8;2)	39
8	39	78*	39	80* 1CB Dmin	39	82*	35	80
9	38	78*	39	40 1CB Dmin ECE	39	39	38	38 Dmin
10	39	40	39	39	39	40	42 Dmin	40 Dmin
11	78*	39	39	39 ECE	39	39	39	101*2Dic(l,l) Rb(l.l)Dic(l,l)
12	40 T(2;14)	40	39	80* Rb(2.2)	39	40	39	39
13	39	41	39	41 Dmin	41	39	38 T(13;8;18)	83*
14	39	41 Dmin	39	35 ECE	39	39	40	39
15	39	39 Rb(5.19)	39	38	39	39	39 T(19;3)	41 ECE Dmin Hsrl
16	39	40	39	36 Dmin	39	39	37 ECE	37
17	39	40	39	41	79	112*	37	39
18	39	39 Dmin	39	39 T(5;l) Rb(5.8) Dmin ECE	36	39	80	80*
19	40 ECE	39 Dmin	39	40 ECE	79	78	39 ECE	39 Dmin
20	41		39	80* ECE	39	Rb(16,16)	39 Rb(3.5) Rb(14.19)	37 Dmin
21	37	78* Dmin	39	40 Rb(Del5.12)		39	37	38 T(Hsrl;13)T(8;18)
22	40	76	39	39 Dmin				
23	72	42	39	40				
24	78	76*	40	80*				
25	39	41 1CB	39	40 Dmin				

CisA: cisatracurium 0.2 µg/kg; CisB: cisatracurium 2.0 µg/kg; PanA: pancuronium 0.1 µg/kg; PanB: pancuronium 1.0 µg/kg; VecA: vecuronium 0.1 µg/kg; VecB: vecuronium 1.0 µg/kg; ProA: propofol 2.0 µg/kg; ProB: propofol 20 µg/kg; ECE, extrachromosomal elements containing chromosomal staining genomic DNA; Dmin, double minute, presence of 1 or more pieces of amplified nonstaining genomic DNA, visualized by the DAPI counter stain; CB, chromatid break representing a break in the chromatin structure; Hsr, homogeneous staining region represents intrachromosomal gene DNA amplification.

^a^Number of images acquired; *endoreduplication, an amplification of genomic DNA leading to chromosomal duplication without subsequent cell division.

In the vecuronium-treated cells with 10×dose, six metaphase images with equal to or greater than 76 chromosomes (6/20) or 30% were present. Chromosome numbers of 76, 78, 80, 82, 98, and 112 chromosomes were noted. Three of these cells displayed the phenomenon of endoreduplication (82, 98, and 112), where mitosis occurs without cell division[13]. There was one new aberration, Rb (16.16). However, the modal chromosomal number remained stable.

**Fig. 1:**
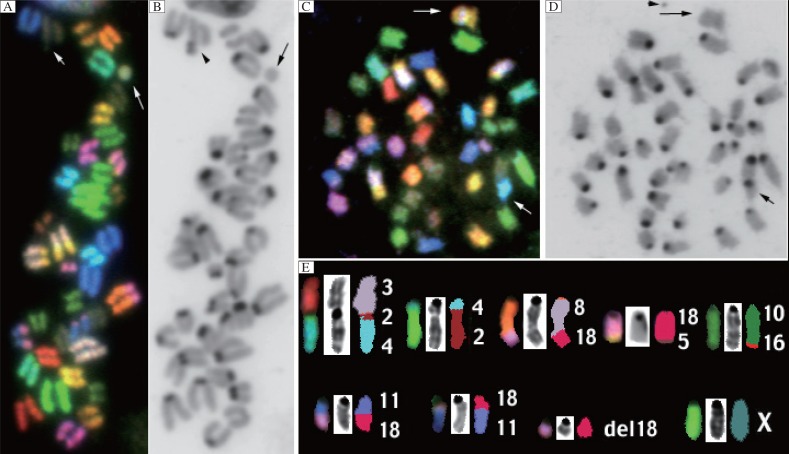
Spectral karyotyping (SKY) of chromosomal aberrations. SKY(A) and inverted DAPI of high dose (1.0 µg/kg) pancuronium (B) with endoreduplication, extrachromosomal elements (ECE) (long arrow), and chromatid breaks (short arrow); SKY(C) and inverted DAPI of low dose (0.2 µg/kg) cisatracurium (D) showing the insertion of chromosome 1 material into chromosome 5 (large arrow) and the T(10;19) centromere-telomere fusion (short arrow). Also present is a Dmin, middle panel D (arrow head); P388 control cell line (E) is consistent with prior chromosomal aberrations (SKY, inverted DAPI, and classification).

At 10× higher dose, pancuronium had a modal number of 40 chromosomes. There were 5/20 metaphases with 78, 80, 80, 80, and 80 chromosomes all displaying endoreduplication. New aberrations were present, including double minute (Dmin) chromosome, 6/25 images, representing amplified gene specific DNA that vary in size and number, and were visualized by DAPI counterstaining[14],[15]. Extrachromosomal elements (ECE) were observed in 7/25 metaphases, which represent amplified chromosomal genes containing DNA visualized by both SKY and DAPI. Chromatid breaks in 3/25 metaphases, and random chromosomal translocations were observed, including nonreciprocal T(5;1) and Rb(2.2), each occurring just once. ***Fig 1A*** and ***1B*** demonstrate high dose pancuronium treated cells with endoreduplication, ECE, and chromatid breaks (cell #3, ***Table 1***).

### Cisatracurium

P388 lymphoma cells treated with clinically equivalent dose of cisatracurium retained the baseline modal number of 39 chromosomes. There were three metaphases with 72, 78, and 78 chromosomes, respectively, with the latter demonstrating endoreduplication. New chromosomal aberrations were identified. One metaphase with ECE was observed. Dmin was present in 5/25 cells and three nonreciprocal translocations T(2;14), T(2;10), and a centromere-telomere translocation, T(10;19), as well as an insertion of chromosome 1 into the body of chromosome 5 yielding Is(1;5), which was present in the same cell as the T(10;19). ***Fig. 1C*** and ***1D*** show Is(1;5) and a centromere-telomere translocation between chromosomes 10 and 19.

The 10×dose resulted in a greater degree of chromosomal numerical variability and the modal number increased to 40 chromosomes, but was only present in 5/25 metaphases. One chromatid break was identified. There were 6/25 metaphases containing 76 or more chromosomes (76, 76, 78, 78, 78, and 78). All but one of the 76 containing metaphases demonstrated endoreduplication. The incidence of endoreduplication in high dose cisatracurium was 24% compared with 4% in clinical dose treatment group. New chromosomal aberrations were present including Dmin in 4/25 images, one nonreciprocal translocation with T(5;19), and one new Robertsonian translocation with Rb(5.19).

### Untreated control

The untreated P388 lymphoma control cell line consisted of the modal number of 39 chromosomes and contained previously noted stable chromosomal rearrangements present in 20/20 images, which are summarized in ***Fig. 1E***. A single Robertsonian chromosome, T(2;4)Rb(2.3) and three reciprocal translocations T(1;13), T(11;18) and T(2;4), were noted. The T(2;4) product underwent an additional centromeric Robertsonian fusion, creating the T(2;4)Rb(2.3) complex chromosome. Additional aberrations included three non-reciprocal translocations T(8;18), T(10;16), and T(18;5), a large central deleted portion of chromosome 18, and monosomy of chromosome X (the tumor was initially induced in a female mouse). These rearrangements were first visualized by SKY in 1999[10] and the P388 leukemic genome has remained stable over the intervening decade despite periodic passages by laboratory personnel.

### Propofol

At clinical dose, the modal number was 39 chromosomes, with 1/21 metaphases containing 80 chromosomes. Chromosomal aberrations occurred in 10/21 metaphases analyzed, including ECEs in 3/21 (14%), Dmin in 2/21 (9.5%), and nonreciprocal translocations and Robertsonian fusions in 5/21 (24%) including Rb(5.16), Rb(14.16), Rb(3.5), Rb(14.19), T(8;2), T(13;8;18), and T(19;3). The Rb(14.16) and T(8;2) occurred in the same cell and the Rb(3.5) and Rb(14.19) also occurred together (***Table 1***).

**Fig. 2:**
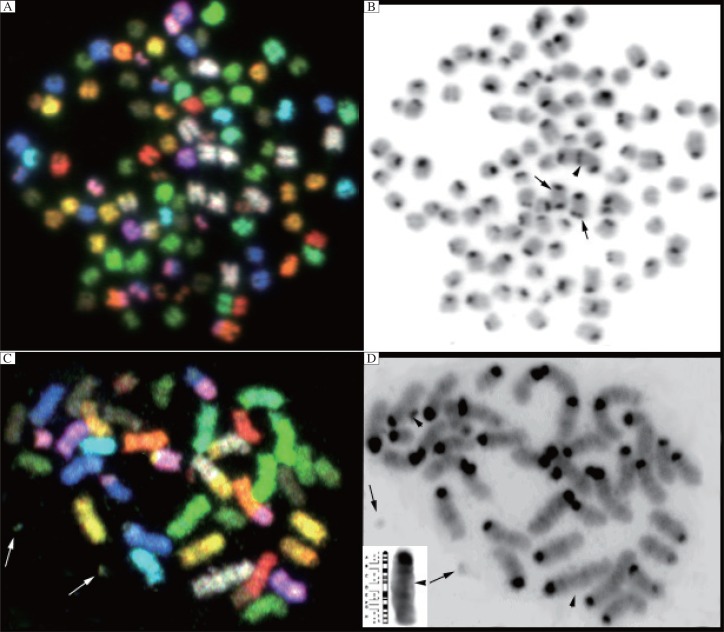
Spectral karyotyping (SKY) of chromosomal alterations in propofol treated cells. SKY(A) and inverted DAPI (B) image from the high dose propofol treatment (20 µg/kg) with endoreduplication. Two dicentric chromosomes: Dic (1;1) (small arrows), and Rb(1.1) Dic(1;1) complex rearrangement visualized best in panel B (arrow head). SKY(C) and, inverted DAPI(D) image from the high dose propofol treatment showing ECE's visualized in both images (two arrows) and the Hsr chromosome 1 band C (arrow head). Insert bottom left of panel D showing increased band C genomic material next to standard mouse chromosome 1 ideogram. The insert is slightly enlarged and enhanced to aid in the interpretation. Arrowhead marks the suspected amplification of the suband region of band C4.

In the 10×dose range, the modal number remained 39 chromosomes with 6/21 identified metaphases having 80 or more chromosomes (80, 80, 80, 83, 84, and 101). Endoreduplication events occurred in 3/22 metaphases (80, 83, and 101). New chromosomal aberrations occurred with Dmin in 6/21 and ECE in 1/21. Nonreciprocal translocation events and Robertsonian translocations include T(8;18), Hsr(1;13), two Dic(1,1), Rb(1.1), Dic(1,1), and Rb(8.10). The two dicentric (Dic1) chromosomes and the Rb(Dic1;1) representing a single metaphase also underwent an endoreduplication event (***Table 1*** ce11 #11, ***Fig. 2A*** and ***2B***). Two metaphases (***Table 1***, cell#15 and 21, ***Fig. 2C*** and ***2D***) demonstrated a homogeneous staining region (HSR) visualized best in the DAPI counterstain within chromosome 1 (***Table 1***, cell #15). This likely represents regions of intrachromosomal duplication, which has been found to represent gene amplification[15],[16].

## DISCUSSION

Genomic instability is now recognized as a hallmark feature in the development of cancer, acting through genetic and epigenetic changes that can result in defective apoptosis[17],[18]. Genomic instability can be further subdivided into chromosomal instability and microsatellite instability. Chromosomal instability is subdivided into structural and numeric chromosomal instability. Numeric chromosomal instability (chromosomal ploidy variability) has been shown to arise from aberrant division, cell fusion, faulty spindle checkpoint fidelity, or failed cell division[17],[19]. Structural chromosomal instability (double strand breaks, dicentrics, chromosomal deletions, translocations, double minutes, extrachromosomal elements, and homogeneously staining regions) results from defective DNA damage repair machinery[17]. In fact, aneuploidy is now being considered as an essential feature of oncogenesis[20]-[23].

We chose the P388 lymphoma cell line to assess genomic instability because of its relatively easy growth requirements and the fact that it has demonstrated stability in culture without undergoing spontaneous structural chromosomal instability. Modest numerical instability in untreated P388 lymphoma cells certainly exists with a range of chromosome numbers from 37-42. However, in two studies a decade apart, the modal number was maintained[10]. In our study, clinically relevant doses of cisatracurium and propofol increased the structural and numeric chromosomal instability of the P388 lymphoma cell line. Vecuronium and pancuronium, primarily benign at clinical concentrations, demonstrated marked numerical chromosomal instability at supratherapeutic doses (***Table 1***). This dose response is similar to the frequency of increased sister chromatid exchanges found in rocuronium treated peripheral blood lymphocytes[5].

Cisatracurium undergoes spontaneous degradation, termed Hofmann elimination, at physiologic pH and temperature[24]. The intermediary products are landaunosine and a monoquaternary acrylate ester[25]. The latter has been implicated in causing anaphylactic reactions[26], increasing oxidative stress[27], inducing apoptosis[28], positive clastogenic and genotoxic effects in cell culture assays[29]-[31], and inducing skin cancers[32]. In our study, both numerical and structural chromosomal instability appears in a dose-effect relationship with a modest increase in the modal number, a 20% increase in the number of metaphases demonstrating endoreduplication, and 50% increase in observed structural chromosomal instability.

Because of its unique mechanism of elimination, cisatracurium is often utilized in patients with hepatic or renal disease[33], and in patients with respiratory failure who develop ventilator dyssynchrony requiring muscle relaxation[34]. *In vivo*, monoquaternary acrylate esters undergo further hydrolysis by non-specific plasma esterases to a monoquaternary alcohol, which can also undergo Hofmann elimination[25]. *In vitro*, no further metabolism can take place, and it cannot be overlooked that the genomic instability uncovered in this study was likely influenced by the fact that rapidly dividing cells were being subjected to prolonged contact with modestly oncogenic acrylate esters. This genome destabilizing effect may be inappreciable in intermittent boluses used during surgery. However, patients in the intensive care unit receiving constant infusions, especially those having recently been treated with chemotherapeutic agents, radiation therapy, immunosuppressive drugs, or have underlying genetic disease predisposing to genomic instability should not receive long term infusions of cisatracurium.

Propofol, because of its reliable pharmacokinetic onset and offset, has become a commonly used agent given as a continuous infusion to provide sedation in patients in whom agitation would be clinically detrimental[35], sedation for procedures both in and outside of the operating room[36],[37], as an induction agent for general anesthesia[38], cerebral protection in central nervous system (CNS) procedures[39], or as an infusion to produce deep anesthesia that can be quickly reversed[40]. However, propofol infusion for prolonged periods (>48 h) and high doses [>5 mg/(kg·h)] can lead to propofol infusion syndrome characterized by metabolic acidosis, lipidemia, hypotension, bradycardia, acute renal failure, and cardiovascular instability[41],[42]. The likely mechanism is thought to occur secondary to inhibition of free fatty acid oxidation[43] that may occur secondary to an underlying carnitine deficiency. There is also an observed inhibition of mitochondrial complex I[44],[45], which can occur at clinical doses[46], leading to depletion of cellular ATP. In our study, cells treated with propofol showed a dose related structural chromosomal instability. Treatment *in vitro* removes the redistribution and metabolism of propofol that would normally occur *in vivo* allowing cultured cells to be potentially overwhelmed by the drug. Propofol induced chromosomal instability may still be relevant in patients with underlying mitochondrial mutations, chromosomal instability syndromes, or concurrently being exposed to chemotherapeutic agents.

Studies that attempted to quantify chromosomal changes between the patient's primary cancer and the subsequent development of metastatic cancer have been performed for both hepatocellular and prostate cancer. Patients with disease relapse, who went on to develop recurrent malignancy, typically show greater diversity of chromosomal changes, especially DNA amplifications (putative gene amplifications) and deletions (loss of tumor suppressor genes)[47],[48]. However, it is unclear as to the role that surgical factors or anesthetic exposure may have had in these malignant changes[49],[50].

An obvious limitation of our study would be the dose-concentration available to the cells. The cell medium is supplemented with FCS, which contains a substantial percentage of albumins[51] available for drug binding. However, free drug levels were not ascertained in this study. Further study limitations include the utilization of a leukemic cell line to examine genomic instability, as such cells may be unable to undergo apoptosis or genomic repair. The period of 6 h for culture was arbitrarily selected to represent a moderate length exposure. A time course study, perhaps evaluating cells up to 48-72 h, may be an important next step. It is possible that chromosomal changes may not be stable, and upon longer exposures lead to cell death or potential genomic repair. Re-culture of previously treated cells can also be performed, and analyzed to see if the new chromosomal changes are inherited after several passages. Further studies, perhaps utilizing human peripheral blood lymphocytes stimulated to divide in the presence of these agents may be able to confirm the importance of these results.
